# Organic Acids in Aquaculture: A Bibliometric Analysis

**DOI:** 10.3390/foods14142512

**Published:** 2025-07-17

**Authors:** Gidelia Araujo Ferreira de Melo, Adriano Carvalho Costa, Matheus Barp Pierozan, Alene Santos Souza, Lessandro do Carmo Lima, Vitória de Vasconcelos Kretschmer, Leandro Pereira Cappato, Elias Marques de Oliveira, Rafael Vilhena Reis Neto, Joel Jorge Nuvunga, Jean Marc Nacife, Mariana Buranelo Egea

**Affiliations:** 1 Department of Science Animal, Federal Institute of Education, Science and Technology Goioano, IF Goiano, Campus Rio Verde, Rodovia Sul Goiana, Km 01, Rio Verde 75901-970, GO, Brazil; gidelia.melo@estudante.ifgoiano.edu.br (G.A.F.d.M.); matheus.pierozan@estudante.ifgoiano.edu.br (M.B.P.); alenesantos47@gmail.com (A.S.S.); lessandro_lima@hotmail.com (L.d.C.L.); vitoria.vasconcelos@estudante.ifgoiano.edu.br (V.d.V.K.); leandro.cappato@ifgoiano.edu.br (L.P.C.); lico.mo12345@gmail.com (E.M.d.O.); jean.nacife@ifgoiano.edu.br (J.M.N.); mariana.egea@ifgoiano.edu.br (M.B.E.); 2 Department of Science Animal, State University Paulista Júlio de Mesquita Filho, Nelson Brihi Badur, 480, Registro 11900-000, SP, Brazil; rafael.vilhena@unesp.br; 3 Center of Excellence in Agri-Food Systems and Nutrition, Eduardo Mondlane University, Julius Nyerere, n° 3453, Maputo P.O. Box 257, Mozambique; joelnuvungak@gmail.com; 4 Faculty of Science and Technology, Joaquim Chissano University, Grande Maputo Street, 88, Maputo P.O. Box 1110, Mozambique

**Keywords:** pathogens, microorganisms, inactivators, antimicrobials, fish farming

## Abstract

Fish production faces various challenges throughout its cycle, from rearing to consumption. Organic acids have emerged as an effective fish feed and meat treatment solution. They promote health and well-being, control pathogens, improve digestion, and contribute to food preservation. This study was therefore carried out to evaluate the evolution of publications on the use of organic acids in aquaculture over time, identifying the leading journals, authors, countries, and relevant organizations associated with the publications and determining the keywords most used in publications and research trends on this type of accommodation using bibliometric analysis. For this analysis, the Web of Science (WoS) and Scopus databases were used, with the keywords and Boolean operators “organic acid*” AND (“pathogens” OR “microorganism*” OR “bacteria” OR “fungi”) AND (“fish” OR “fry” OR “pisciculture”). Ninety-six articles were found in 44 journals, with the participation of 426 authors and 188 institutions, from 1995 to 2024. The most crucial publication source with the highest impact factor was the journal Aquaculture, with 14 articles, 2 of which were written by the most relevant author, Koh C., who received the highest number of citations and had the highest impact factor among the 426 authors. China had the most scientific production, with 26 publications on organic acids in aquaculture. However, Malaysia was the country that published the most cited documents, a total of 386. The most relevant affiliation was the University of Sains Malaysia, which participated in the publication of eight articles. The 10 most frequent keywords were fish, organic acids, citric acid, article, bacteria, growth, microorganisms, *Oncorhynchus mykiss*, animals, and digestibility. The results indicate increased publications on the benefits of using organic acids in aquaculture, highlighting their effectiveness as antibacterial agents and promoters of zootechnical development. However, gaps still require more in-depth research into the ideal dosages, mechanisms of action, and long-term impacts of these compounds.

## 1. Introduction

Aquaculture, a method used to produce food and other commercial products, has seen significant growth in recent decades, with its products increasingly appearing on consumers’ tables due to its meat’s nutritional and sensory qualities. Fish consumption will increase by 15 percent by 2030, reaching 21.4 kg per capita, driven mainly by new dietary trends with special attention to improving health and nutrition [1]. These foods are rich in essential fatty acids, bioavailable minerals, and vitamins, while also having lower levels of saturated fat than other animal protein sources [2].

In aquaculture, several challenges emerge during production. One of the most pressing concerns is disease prevention and control, as outbreaks can severely impact fish survival and well-being [3]. The use of antibiotics in aquaculture can lead to adverse effects, including risks to human health due to potential antibiotic residues in fish or the emergence of resistant bacteria [4]. Consequently, the excessive use of antibiotics drives antimicrobial resistance (AMR) [5].

The use of antibiotics has been a widely adopted strategy to prevent diseases and enhance production performance in aquaculture, contributing to fish health and farming efficiency [6]. However, their extensive use presents limitations due to the rise of bacterial resistance and potential risks to human health, which led to the establishment of regulations governing their use in the European Union [7,8,9].

In addition to challenges related to fish health, aquaculture requires continuous improvement in production performance, which is often affected by nutritional and health imbalances [10]. At the same time, reducing environmental impacts is essential, particularly in systems that depend on intensive input use and require proper waste management [11].

Another key challenge in aquaculture is product contamination by pathogens, a problem intensified by the increasing consumption of raw aquaculture products, partly driven by the global spread of oriental cuisine. This consumption trend continues to rise, with various species used in dish preparation. Since these products are typically consumed fresh without heat treatment, infected fish can act as hosts and sources of pathogenic microorganism contamination [12].

Aquaculture growth drives resistance mechanisms similar to those in agriculture, as antibiotics exert selective pressure, accelerating the development and spread of resistance genes (ARGs). Co-resistance in zoonotic pathogens, effective against multiple antibiotics, has become a significant concern. The lack of consistent data on antibiotic usage hinders a precise assessment of the impact of aquaculture and agriculture on resistance. Furthermore, water serves as a medium for the dispersion of drug residues, pathogens, and resistance genes, making aquaculture an increasing threat, especially with the rising demand for seafood [13].

Contamination in aquaculture primarily occurs through the direct use of antibiotics to prevent diseases and enhance fish production and through indirect transmission due to the improper use of veterinary and human antibiotics. Emerging contaminants, such as pharmaceuticals from human activities near aquaculture sites, also impact the environment. Water from rivers, coastal areas, or underground sources, commonly used in aquaculture, facilitates cross-contamination. However, global data on this issue remains scarce [14].

Studies on different zoonotic microorganisms in fish have been carried out, since species contamination can come from the natural microbiota or be transmitted during the handling process, both by microorganisms that cause spoilage and by significant pathogens such as *Escherichia coli*, *Salmonella* spp., *Listeria monocytogenes*, and *Staphylococcus aureus*, which are often associated with food outbreaks [15,16].

Adding organic acids, either in combination—such as acetic and ascorbic acids—or individually, like lactobionic acid, during storage can compromise the integrity of the bacterial cell membrane, leading to intracellular protein leakage and increased conductivity [17]. Additionally, these compounds lower the pH of the fish, creating an unfavorable environment for bacterial growth [18], thereby extending its shelf life. Nebulization or immersion treatments with lactic and peracetic acid have also shown great potential in fillet processing, preserving appearance, and enhancing meat quality [16]. Several feed additives, such as organic acids and essential oils [19], probiotics [20], prebiotics [21], and amino acids [22], are being used as alternatives to antibiotics in fish diets, with organic acids standing out among them. Including organic acids in fish diets has also been shown to improve digestibility, mineral absorption, and the activation of digestive enzymes, leading to better growth and feed efficiency [23].

Organic acids, widely used for their preservative, acidifying, and flavoring properties, have demonstrated outstanding potential in aquaculture. Their antimicrobial efficacy not only enhances food safety for consumers but also serves as an effective strategy for disease prevention in production systems. A notable example is the use of fumaric acid as a feed supplement for juvenile Nile tilapia, which has been shown to improve growth performance, feed efficiency, and protein conversion rate while also inhibiting the growth of enterobacteria in the intestine and promoting a healthier microbiota [24]. Other organic acids considered postbiotics, especially short-chain fatty acids (SCFAs) such as propionic, butyric, and acetic acids, play an essential role in enhancing antimicrobial activity and gut health in aquaculture, a rapidly expanding industry closely connected to ecosystem health. These SCFAs significantly influence intestinal physiological and digestive functions, supporting overall fish health [25,26].

Replacing antibiotics in fish feed with acidifiers helps control harmful bacteria, promotes a healthy intestinal microbiome, and strengthens the immune system. Additionally, acidifiers enhance nutrient utilization by improving mineral absorption and supporting digestive tract development, leading to more efficient and sustainable aquaculture production. In addition to enhancing feed digestibility, acidifiers improve mineral absorption and optimize feed efficiency by supporting the development of the digestive tract, contributing to more efficient and sustainable aquaculture production [27].

The main objective of using these compounds is to reduce pH, a favorable condition for activating pepsin, an essential enzyme for protein digestion [28]. Dietary supplementation of organic acids also stimulates the bactericidal activities of lysozyme and serum [29]. Furthermore, acidifiers promote an increase in the length of intestinal villi, which is related to a greater capacity for nutrient absorption in the intestinal lumen [30]. Incorporating these additives into the diet improves the ability of fish to absorb nutrients and maximizes feed intake efficiency [27].

Katya et al. [31] the efficacy of organic acid mixtures as substitutes for antibiotics in the diet of olive flounder (*Paralichthys olivaceus*) was evaluated. The fish were fed either antibiotics (oxytetracycline) or mixtures of organic acids. The results showed that the group fed with organic acids had intestinal bacterial counts and *Vibrio* sp. levels similar to those of the antibiotic-fed group. Additionally, these groups exhibited lower cumulative mortality after being challenged with *Edwardsiella tarda*. These findings support the idea that organic acid mixtures are an effective alternative to antibiotics, promoting intestinal health and disease resistance in marine fish and reinforcing their potential for sustainable and preventive management in aquaculture.

Among other applications that the literature has reported for organic acids is their application to sanitizing utensils and fish flesh, using peracetic, citric, and lactic acid compounds. Studies indicate that these acids can significantly reduce the load of deteriorating microorganisms in gutted fish, prolonging the product’s shelf life and guaranteeing its quality [32,33]. Organic acids also contribute to pollutant removal and bioremediation of contaminated sediments. In studies using sediment microbial fuel cells (SMFCs), the degradation of polycyclic aromatic hydrocarbons (PAHs) was enhanced by producing organic acids from starch deposited in aquaculture sediments. Analysis of the accumulation of these compounds revealed a significant positive correlation between electricity generation and PAH removal, suggesting that microbial metabolism driven by organic acids can optimize the decontamination process. However, an excess of these acids lowered the anode pH, compromising microbial activity and inhibiting the efficient utilization of the co-substrate [34].

A bibliometric analysis of using organic acids in food preservation and fish meat sanitation is essential for mapping scientific progress and identifying trends and strategic opportunities in knowledge. It makes it possible to understand the evolution of publications, highlight the prominent journals, authors, institutions, and countries involved, and identify the most used keywords and emerging themes [35]. With a broad view of the scientific landscape through bibliometric analysis, it is possible to direct future research, contribute to the interaction between academia and the market, and promote more effective solutions. Therefore, this study aims to carry out a bibliometric analysis of the use of organic acids in aquaculture, evaluating the evolution of publications, identifying the prominent journals, authors, countries, and relevant institutions, and determining the keywords most used in publications and research trends.

## 2. Methodology

This study used two databases to consult articles published on the use of organic acids in aquaculture. Despite the significant growth in bibliographic data sources and metrics over the last decade, the Web of Science (WoS) and Scopus databases remain the primary and most comprehensive sources of publication metadata and impact indicators. They are essential tools for journal and literature selection, monitoring individual careers, large-scale bibliometric analyses, and research evaluations at all levels [36].

**Figure 1:**
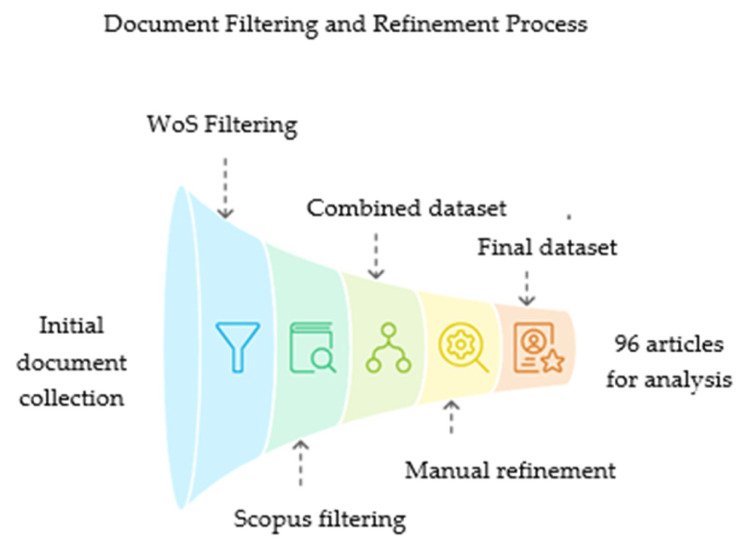
Flowchart of document selection for bibliometric analysis of the use of organic acids in aquaculture.

The simplified methodology for selecting articles can be seen in the flowchart below (Figure 1).

Through the CAPES platform, documents were retrieved from the Web of Science (WoS) and Scopus (DBs) using the keywords “organic acid*,” “pathogens,” “microorganism*,” “bacteria,” “fungi,” “fish,” “fry,” and “pisciculture.” The keyword selection was driven by the aim to encompass the broadest range of studies related to the antimicrobial action of organic acids in aquaculture contexts, along with the Boolean operators “AND” and “OR.” Broader terms were prioritized to avoid premature exclusion of studies that might use varied terminologies across species or research focuses. More specific descriptors, such as “aquaculture feed additives” or “fish health promoters,” were considered; however, they were not adopted due to their narrower scope and potential to limit the number of retrievable articles significantly. In the first database, WoS, 280 documents were found and subsequently filtered to ensure that only scientific articles were selected. After applying the “articles” and “search in all fields” filters, 248 documents remained. Subsequently, 11 procedural articles, 3 early access articles, and 18 review articles were excluded. In Scopus, 299 documents were identified using the exact keywords. The filters used in this database were “articles” and “search within article title, abstract, and keywords” to select only works evaluated by the most rigorous scientific screening. After filtering, 23 analyses, 14 conference papers, 11 book chapters, and 1 book were excluded, leaving 250 articles for review.

The data from 498 articles were exported in BibTeX format and then processed in R to generate a unique dataset. Using Biblioshiny within the Bibliometrix package [37] in R software (version 4.3.2) [38] the dataset was analyzed, followed by an additional manual filtering step, in which the methodologies of all articles were reviewed to exclude those that, although applied in aquaculture, were not related to the use of organic acids.

Among the articles excluded were those that focused on organic salts rather than organic acids, studies carried out on chickens, pigs, or other animals not related to aquaculture, and those that involved probiotic bacteria derived from acids rather than the direct application of the acids themselves. After this filtering process, 55 articles remained from Scopus and 59 from WoS. These documents were selected again based on their titles and exported in BibTeX format for further analysis. The R program was used to identify and remove duplicate documents, excluding 7 duplicates, and create a new database with 107 articles. As some duplicates were not automatically recognized by the program, a manual review was carried out in R to exclude articles with the same DOI (Digital Object Identifier), leaving a final set of 96 articles for bibliometric analysis.

## 3. Results and Discussion

### 3.1. General Analysis

In all, 96 documents were analyzed based on the parameters described in the methodology. These articles were produced in collaboration with 426 authors and published in 44 sources. The annual growth rate over 29 years was 8.26% from 1995 to 2024, with an average of 22.43 citations per document. This growth followed the average growth rate of 6.7% per year from 1990 to 2020, totaling a growth of 609% [39].

Table 1 shows the information about the obtained data. Of the 96 analyzed documents, 1 article was published with early access, and 2 are procedural documents.

**Figure 2:**
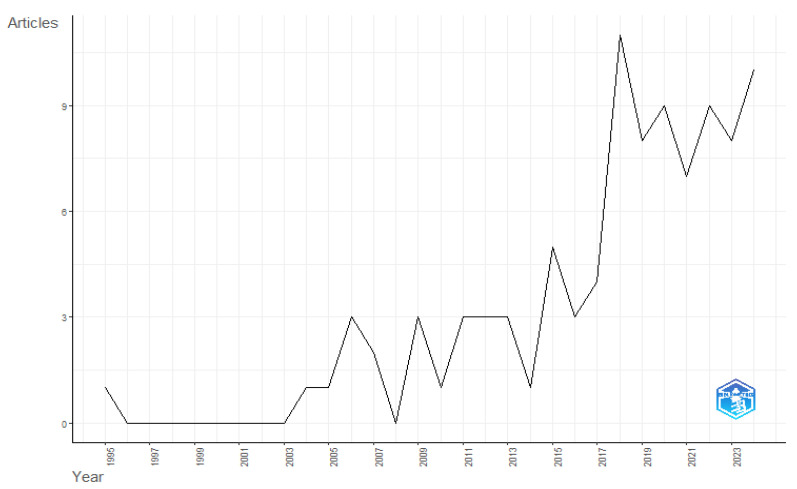
Annual scientific production on the use of organic acids in aquaculture from 1995–2024.

Scientific production using organic acids in aquaculture has increased significantly since 2017, as seen in Figure 2. The expansion of scientific production using organic acids in aquaculture can be attributed to two main factors. The first factor is related to the increasing restriction on the use of antibiotics in fish feed, a highly relevant issue due to concerns about antimicrobial resistance, environmental impacts, and food safety. This concern has driven a significant increase in research on safer and more sustainable alternatives. Various countries and regulatory boards have established strict guidelines for using antibiotics in aquaculture, encouraging the adoption of innovative strategies such as using organic acids, probiotics, and other functional additives [8,9,40]. The second factor is the growing global demand for fish and other aquatic foods, leading to rapid changes in the fishing and aquaculture sectors. Projections indicate a 15% increase in consumption, reaching 21.4 kg per capita by 2030 (as previously described). This growth is driven by rising incomes, urbanization, post-harvest improvements, distribution practices focused on sustainability, and new dietary trends prioritizing health and nutrition [1].

The average of 22.43 citations per document over the analyzed period reflects the scientific relevance and academic impact of studies on using organic acids in aquaculture. This number indicates that the topic has been widely discussed within the scientific community, reinforcing its importance for the aquaculture sector and its contribution to advancing knowledge.

Additionally, the average document age of 6.45 years suggests that the studies are relatively recent but already demonstrate a consolidated citation cycle, indicating a balance between established research and discoveries. This data highlights that research on organic acids in aquaculture is continuously evolving, keeping pace with the growing demand for sustainable alternatives in fish nutrition and regulatory changes in the sector.

The 14.58 percentage of international co-authorships indicates that research on the use of organic acids in aquaculture has a relatively low level of global collaboration, suggesting that national or regional research groups conduct most publications.

The co-authors per document metric (5.79) shows that, on average, each article on the use of organic acids in aquaculture was produced by approximately six authors. This number suggests that even if the level of global collaboration is low, there is a moderate to high level of scientific collaboration, reflecting the complexity of the topic and the need for multidisciplinary expertise.

### 3.2. Most Relevant Sources

Publications related to the use of organic acids in aquaculture totaled 96 documents from 44 different sources. The primary source of these publications was the Aquaculture journal, with 14 published articles representing approximately 14.58% of the documents analyzed. This result was expected, as it is a specialized journal in aquaculture science, widely recognized as one of the leading scientific references with the highest impact in the field. Additionally, its open-access publishing policy facilitates article submissions by researchers from various institutions, contributing to more publications on the topic.

**Figure 3:**
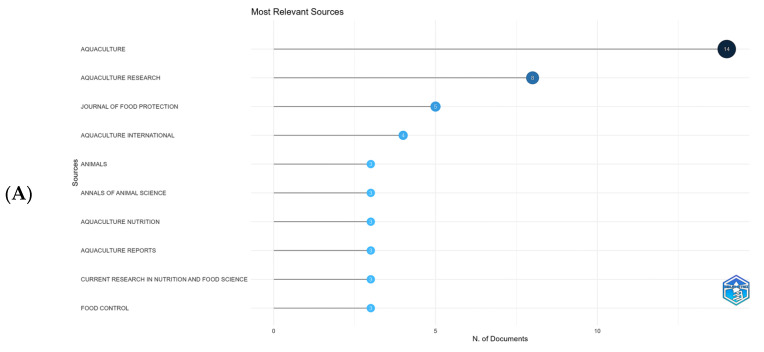

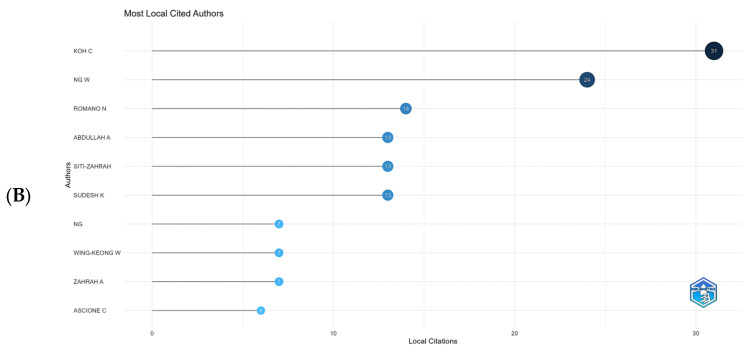
The 10 most relevant sources in the production of articles (**A**) and the 10 most cited authors (**B**) of the analyzed data from the bibliometric analysis of the use of organic acids in aquaculture.

The Aquaculture Research (8.33%) and Journal of Food Protection (5.20%) journals were in second (publishing eight manuscripts) and third (publishing five manuscripts) place, respectively (Figure 3A). Aquaculture International was in fourth place with four publications (4.16%). The fifth most relevant journal was Animals, followed by Annals of Animal Science, Aquaculture Nutrition, Aquaculture Reports, Current Research in Nutrition and Food Science, and Food Control, each with three published articles and a percentage of approximately 3.12 each.

Analyzing the impact factor using the H-index showed a similarity between the most relevant sources of publications and the most impactful sources. However, some journals lost positions compared to their previous rankings as the most relevant sources. This reduction in the H-index of journals is explained by this indicator, considering the total number of publications and the number of submissions received for each article. Thus, the H-index drops when a journal has many publications but few citations to each article. Another explanation is that when a journal receives many citations, they are distributed unevenly among articles. Some articles are highly cited, while others receive few or no citations [41].

For example, Aquaculture has 14 articles published on organic acids in aquaculture over 30 years, the most significant number of publications among the journals, a high total citation rate, distributed unevenly. The most cited article was that of Romano et al. [42] with 116 citations, followed by Ng et al. [43] with 55, Davies et al. [44] with 34 Liu et al. [45] with 25, Libanori et al. [46] with 15, dos Santos et al. [47] with 12, Ohtani et al. [48] with 10, and Suehs et al. [49] with 4, thus confirming a reduction in the number of citations of the other documents until Naya-Català et al. [50] with 2 citations. There is a decreasing trend in citations as the publication date approaches the present, reflecting the shorter exposure time of more recent articles.

On the other hand, Aquaculture Research had only eight articles published over 30 years on using organic acids in fish. Its main article, published by Ng et al. [43], was cited 146 times, followed by Koh et al. [51] with 69 citations, Marchand et al. [52] with 38, Chen et al. [53] with 36, Katya, Park, Bharadwaj, Browdy, Vazquez-Anon, and Bai [31] with 23, and Yilmaz et al. [54] with 21, also confirming that the most recent documents were less cited. Still, among the eight articles, all had more than eight citations; in Aquaculture, not all 14 had 14 citations. The results of the H-index impact factor of the 10 most impactful sources among the 44 journals published on organic acids in aquaculture can be seen in Table 2.

The H-index is the largest number h of published articles that have each received at least h citations [55]. For example, Aquaculture Research has an H-index of 8, meaning at least eight articles published in that journal received eight or more citations each. In contrast, Aquaculture has an H-index of seven. The metric combines quantity and impact of publications and is widely used to compare scientific productivity objectively [56,57]. Although concerns have been raised regarding self-citations, the robustness of the H-index against their influence is now better understood—it cannot be increased by self-citations [56].

Among the sources analyzed, the journal Aquaculture (h = 7) published more papers. However, its H-index was lower than Aquaculture Research’s (h = 8), placing it second in the impact factor. This behavior can be explained by the fact that it received fewer citations than the first-placed journal in the ranking and that less time had elapsed since its first publication in the database searched. PY Start identifies the year in which the first article on organic acids in aquaculture was published in the databases researched. Therefore, journals that began publishing on the topic more recently may have a lower impact factor due to less time since the first publication and fewer years to cite this document.

The highest impact factor was found for Aquaculture Research, which had 373 citations from eight published documents. The third and fourth positions were occupied by the Journal of Food Protection and the journal Aquaculture International, respectively. The former had an impact factor and number of publications of five, and the latter had an H-index of four and four publications. While the Journal of Food Protection was cited 90 times, Aquaculture International received 57 citations.

In fifth place was Aquaculture Reports, followed by the International Journal of Food Microbiology, the Journal of Food Science, and PeerJ, with three publications each and an impact factor h = 3, distinguished by the initial year of publication and number of citations (Table 2). After calculating the impact factor H, the journal Animals occupied the tenth position, previously occupied by Food Control.

Another distinction observed relates to the Annals of Animal Science, Food Control, Current Research in Nutrition and Food Science, and Aquaculture Nutrition, which, despite being among the top 10 sources, did not stand out among the 10 most impactful sources when the number of citations was considered (Table 2).

All journals described in Table 2 are peer-reviewed. Still, Aquaculture Research, the Journal of Food Protection, Aquaculture Reports, PeerJ, Animals, and Aquaculture Nutrition only publish open access, and Aquaculture, Aquaculture International, the International Journal of Food Microbiology, and the Journal of Food Science are hybrid and support open access. Open access allows other researchers and universities to access the manuscripts free of charge, popularizing the information and science in general [58].

### 3.3. Most Relevant Authors

**Figure 4:**
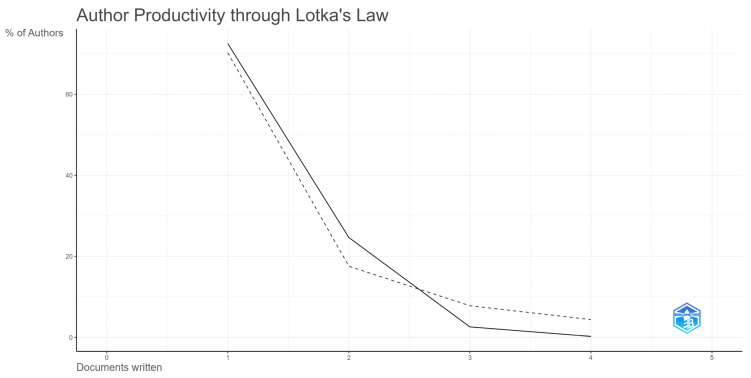
Author productivity using Lotka’s Law of analyzed using data from the bibliometric analysis of organic acids in aquaculture. The dotted line indicates the theoretical distribution per Lotka’s Law, whereas the solid line depicts the distribution observed in the analyzed dataset.

Over the last 29 years, 426 authors have published 96 articles on the use of organic acids. These researchers have received 275 local citations and have been cited 11,427 times. Of the 426 authors, 31 were not cited; the most cited, Koh C., received 386 global and 31 local citations. Data on the 10 most cited local authors is shown in Figure 4, and the 10 authors with the highest global impact index are shown in Table 3.

Local citations can be understood as those made by documents within the specific dataset, such as the community of authors and documents appearing in the bibliometric analysis. In comparison, global citations refer to the number of times a document was cited across the entire database, in the case of this WoS and Scopus article, regardless of the published topic. Within the community, the author Koh C. received the highest number of citations (31) and was also the most cited author overall, with 386 citations and the highest impact index (h = 4) from four articles (Figure 3B). Aubourg S. was the author with the second most relevant impact index (h = 3), receiving 81 citations from published documents; however, within the community, he dropped to 44th position, with only 1 citation.

In respective order from third to ninth place were Martins M., Meinelt T., Ng W., Pereira S., Sahu N., Santos G., and Yilmaz S., all with three publications each and an impact factor h equal to 3. Among the seven authors, the one in fifth place by impact factor index received 317 citations, and the one in ninth place received 130. Abedian K. A. had the tenth highest impact factor, h = 2, 2 publications, and 57 citations.

The 10 most relevant authors published between three and four articles each and 1053 citations, approximately 9.21% of the total global citations, represented by only 2.35% of the authors. The timeline showed that the first article on using organic acids in aquaculture was published in 1995, while the most relevant authors published their article in 2006 (Table 3).

Some events that occurred during this period may have affected this growth, such as (i) the growth of the sector that was accentuated during this period (as discussed previously); (ii) the 1990s saw a global increase in interest in alternatives to antibiotics in animal feed, including aquaculture, due to concerns about antimicrobial resistance [59]; (iii) the ban on the use of antibiotics as growth promoters in Europe in 1995 and their complete ban in 2006 [60]; (iv) the occurrence of scientific events and environmental policies related to food security, such as the United Nations Conference on Environment and Development (ECO-92) in 1992, which highlighted sustainable development and responsible management of natural resources [61] and the Mercosur Aquaculture Symposium (AQUIMERCO) in 2004, which brought to light a large amount of scientific work using organic acids [62]; and (v) the adoption of practices such as the FAO Code of Conduct for Responsible Fisheries in 1995, which established international guidelines for sustainable fisheries and aquaculture [63].

The author with the most publications, Koh C, published one article in 2009 [51], two in 2015, and one in 2016, totaling four publications and 386 citations, representing approximately 36.66% of the 10 most cited authors (386/1053). According to Table 3, he also had the highest impact index H (h = 4), contributing to the publication of four documents on the subject, all from his affiliated institution, Universiti Sains Malaysia (USM), which had the highest relevance of all the institutions. In addition, his country, Malaysia, published 11 documents and received the highest number of citations. By quickly analyzing the author’s professional network (LinkedIn^®^), it was possible to identify the author’s departure as a researcher from the University in 2015. He integrated into the company Cargill Aqua Nutrition (CQN), where he held the position of Technology Application Manager until 2020, and, from that year to the present, he has held the position of Technology Application Lead and Nutritionist.

The author Aubourg S., affiliated with Spain’s Marine Research Institute, published two articles in 2012 and one article in 2013, on a par with the other authors, who also published three articles over the years (Table 3) and each of whom accounted for 3.12% of the documents published. The 10 most relevant authors account for 32.29% of the 96 articles analyzed.

The number of articles published by the authors in this time interval ranged from one to four, as shown in Table 4. The productivity of authors writing about using organic acids in aquaculture follows Lotka’s Law (Figure 4), which suggests that some authors are highly productive. At the same time, the majority publish only one or a few articles. Only one author (0.23%) participated in publishing four articles. In contrast, 309 authors (72.53%) participated in the publication of just one article, 105 authors (24.64%) participated in the publication of two articles, and 11 authors (2.58%) participated in the publication of three articles.

### 3.4. Most Relevant Articles

Knowing the primary documents published in a given area can guide researchers and institutions. Analyzing these documents makes planning new research based on advancing scientific knowledge possible since they highlight widely accepted themes and emerging trends portrayed in frequently referenced articles.

**Figure 5:**
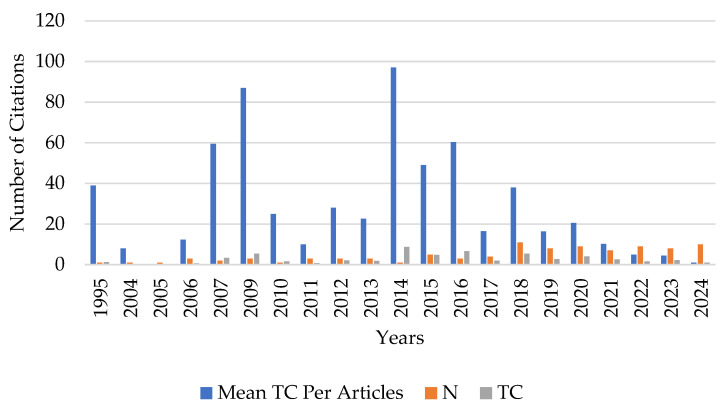
Average citations per article and year of analyzed data from the bibliometric analysis of using organic acids in aquaculture. N: number of articles published and TC: total number of citations.

The relevance of the articles published on using organic acids in aquaculture was analyzed by the number of citations received and the time of publication. The 96 analyzed documents were cited 2157 times over 29 years, showing variability. 2014 had the highest average annual citation per article (Figure 5), with 22 articles published up to that year. This indicates that the studies published up to that period significantly impacted the scientific community, serving as a foundation for subsequent research and consolidating knowledge on using organic acids in aquaculture. The peak observed in the average number of citations per article in 2014 can be explained by the fact that only one article was published that year, which accumulated 97 citations over 11 years, resulting in an annual average of 8.82. Since the average is calculated by the total citations divided by the number of articles published, a single highly cited article significantly increases this value.

**Figure 6:**
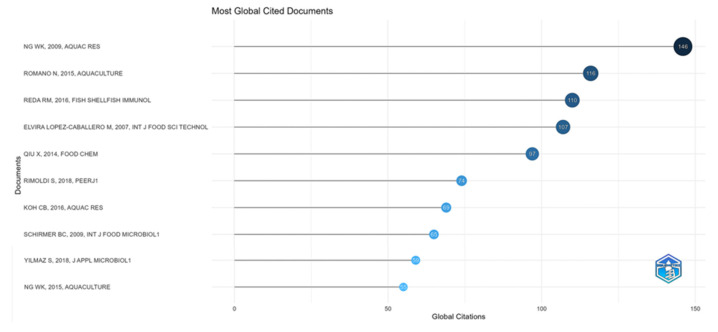
The 10 most globally cited documents of analyzed data from the bibliometric analysis of using organic acids in aquaculture [42,43,51,54,64,65,66,67,68,69].

Among the most globally cited documents, the article published by Ng W. K. and collaborators [64] in the journal Aquaculture Research leads with 146 citations over 15 years (Figure 6). The second most referenced document was published in 2015 by Romano N. and collaborators in the journal Aquaculture, receiving 116 citations in the 9 years since its publication. Both authors are affiliated with Universiti Sains Malaysia, and the first is listed as a co-author in the second’s work, the same paper published in 2015. Both the publication sources and the authors’ affiliations stood out as the most relevant in this bibliometric analysis, playing a key role in the notoriety of these articles.

The third most cited document received 110 citations over 8 years and was published in the journal Fish Shellfish Immunol in 2016 by Reda R. M. and collaborators [65]. Next, the fourth most cited document globally was the last one to receive more than 100citations; it was published in 2007 by Elvira Lopez-Caballero M. [66] in the International Journal of Food Science & Technology.

The three most cited articles are about using organic acids in fish feed [42,64,65], but articles with this theme total 6 among the 10 most cited [42,43,51,64,65,67]. All studies focus on nutrition and health in aquaculture, emphasizing replacing antibiotics with natural additives such as organic acids and probiotics that improve digestion, immunity, the growth of aquatic organisms, and the reduction of bacterial infections. This reinforces the potential of these substances as sustainable alternatives to antibiotics in aquaculture.

These studies reported improvements in the growth and intestinal health of fish and shrimp, reducing the presence of pathogens and promoting the growth of beneficial microorganisms [51,64,65,67] that probably impacted the immunity of the animals [42,43], with modest effects on the growth of the species [42,64], most likely by demonstrating improved digestibility in the animals [43] by increasing feed efficiency [65].

Thus, these manuscripts demonstrate that organic acids (i) can act as natural antimicrobial agents, but their effect depends on the dosage and the species of fish and/or shrimp; (ii) by acting as synthetic growth promoters; (iii) by modulating the microbiota of aquatic organisms; and (iv) by improving the performance and resistance of fish in aquaculture. All these characteristics contribute to the drive for more scientific studies and indirectly to using organic acids, making aquaculture more sustainable.

Three articles studied organic acids as an agent that promotes increased shelf life of fish meat for food [66,67,68,69]. The studies showed that, although the application technique and often the type of acid were different, the results demonstrated a decrease in microbial growth and lipid oxidation processes, which resulted in better sensory characteristics of the meat.

Finally, only one manuscript evaluated organic acids against microorganisms in the fish microbiota. Yilmaz et al. [54] evaluated different natural additives and modified packaging to extend the shelf life of seafood. They demonstrated that compounds such as organic acids have antimicrobial and antioxidant activity, reinforcing and explaining the behavior of these acids when applied directly to the meat [68,69].

The Digital Object Identifier (DOI) for each of these 10 documents can be found in Table 5.

### 3.5. Words

**Figure 7:**
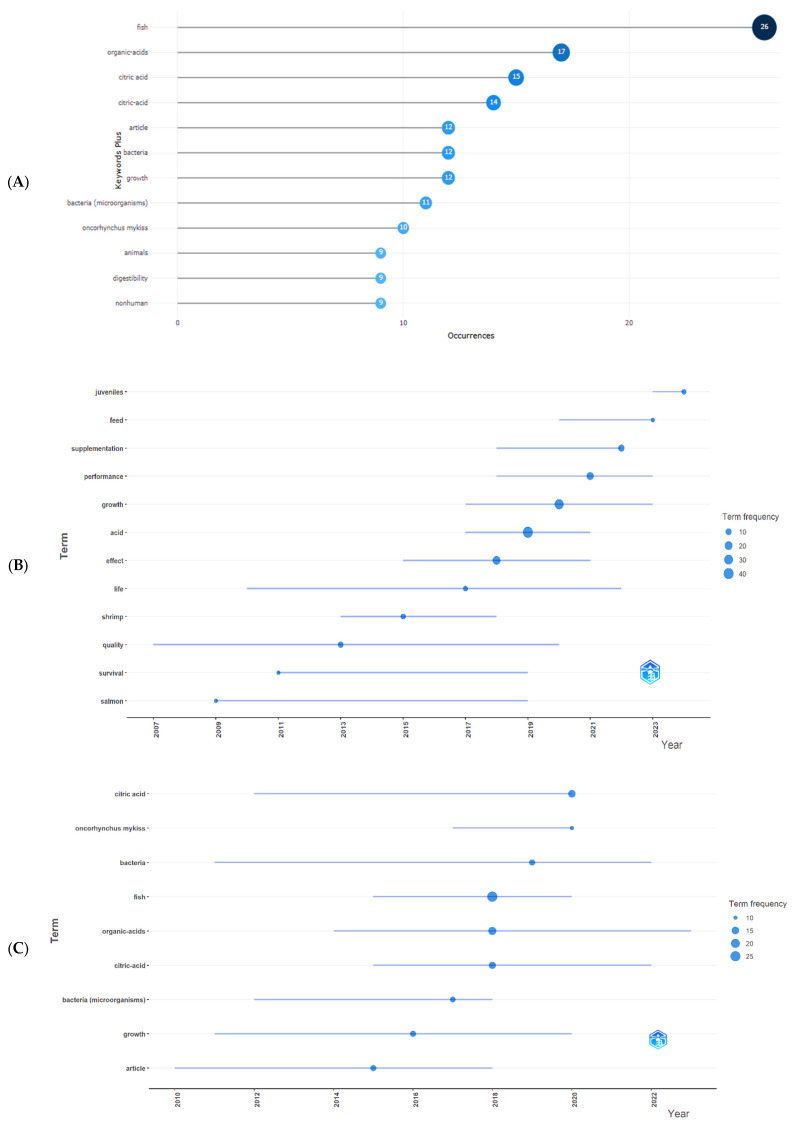
The 10 most cited words in the documents (**A**), topics of trending words that appear most frequently in the title (**B**), and trending topics of plus words (**C**) of the analyzed data from the bibliometric analysis of the use of organic acids in aquaculture.

Highlighting the most recurring words in the writing of documents allows for the identification of methods and concepts that have attracted the attention of researchers. The relevance of these words aids in understanding trends and scientific priorities in a given area of knowledge, enabling the development of strategies and decision-making for guiding research. The 10 words mentioned most frequently were fish, organic acids, citric acid/citric-acid, article, bacteria, growth, bacteria (microorganisms), *Oncorhynchus mykiss*, animals, digestibility, and nonhuman. The frequency with which these words appear in the documents can be seen in Figure 7A.

It is observed that the expression “citric acid” was written in two ways in the documents, with and without the use of a hyphen. Therefore, if the two forms are considered a single word, it is the most cited word in the articles (29 times). Citric acid is an efficient dietary supplement that promotes greater weight gain, specific growth rate, protein efficiency rate, and lower feed conversion rate, improving digestibility and increasing survival [70,71]. It can also mitigate oxidative damage and balance the intestinal microbiome [53]. Yildiz and Yilmaz [72] also found interesting results indicating that adding citric acid to the diet can exert a sanitizing effect on the cultivation environment, promoting the reduction of infectious forms of microorganisms present in the water.

The word bacteria also appears with two classifications, one as a standalone (12 times) and another followed by the word “microorganisms” (11 times). If both are considered for their unique meaning, this will rise to third among the words mentioned most frequently. Microbial deterioration is a worrying factor in the preservation of products from aquaculture, as they are highly perishable foods due to their high water content. Inhibiting the growth of microorganisms at all stages of production ensures a contaminant-free product for the consumer and reduces losses for the producer. The use of organic acids, such as citric and peracetic acids, is effective in extending the shelf life of refrigerated fish, as reported by Ntzimani, Semenoglou, Dermesonlouoglou, Tsironi, and Taoukis [33], promoting an increase of 45.5% and 63.6% in the shelf life of the products, respectively. Organic acids have bacteriostatic capacity due to their ability to permeate the bacterial cell membrane in their undissociated form and, once inside the bacteria, release H^+^ ions, acidifying the cytoplasmic pH. This consequently destabilizes bacterial metabolism, directly interrupting the normal physiology of the cytoplasm, including protein replication and synthesis, causing bacterial death [73].

With the results of the analysis of the documents and keywords, it was possible to identify the trending topics of words that appear most frequently in the titles and keywords. Observing Figure 7B, the three words with the highest trend are “acid,” with a frequency of 41, followed by “organic,” frequency = 30, and “growth,” frequency = 31. When analyzing the plus words (Figure 7C), this panorama changes, with “fish” having the highest frequency (26), followed by “organic acids” with 17, and “citric acid” with 15. It is worth noting that, in the bibliometric analysis, the terminology referring to citric acid presented a lexical variation not recognized by the interface used, resulting in the distinction between “citric acid” and “citric-acid” (Figure 7A). Although both forms refer to the same compound, “citric acid” appears 15 times, while “citric-acid” appears 14 times among the plus keywords. This variation stems from the automated data processing and has been retained to preserve the fidelity of the analysis.

### 3.6. Most Relevant Countries

Thirty-five countries participated in the publications on the use of organic acids in aquaculture. Table 6 shows the 10 countries that produced the most articles from 1995 to 2024, including their scientific contributions and co-authorships. China stood out as the most productive country on the subject (26 articles), followed by Iran (25 articles), the United States (21 articles), and Brazil and Turkey (20 articles).

China’s prominence in scientific production can be attributed to its large population and food traditions, which place it as the third largest importer of fish for its domestic consumption and processing industry [1]. In addition, the country benefits from strong political support, as evidenced by the scale of its aquaculture sector. According to Yue et al. [74], Chinese aquaculture is expected to remain stable, sustainable, and competitive both nationally and internationally, driven by proactive public policies, strategic planning, emerging technologies, scientifically designed production systems, and the growing global demand for aquaculture products. This country has become the world’s leading producer, exporter, and processor of aquatic products [75].

**Figure 8:**
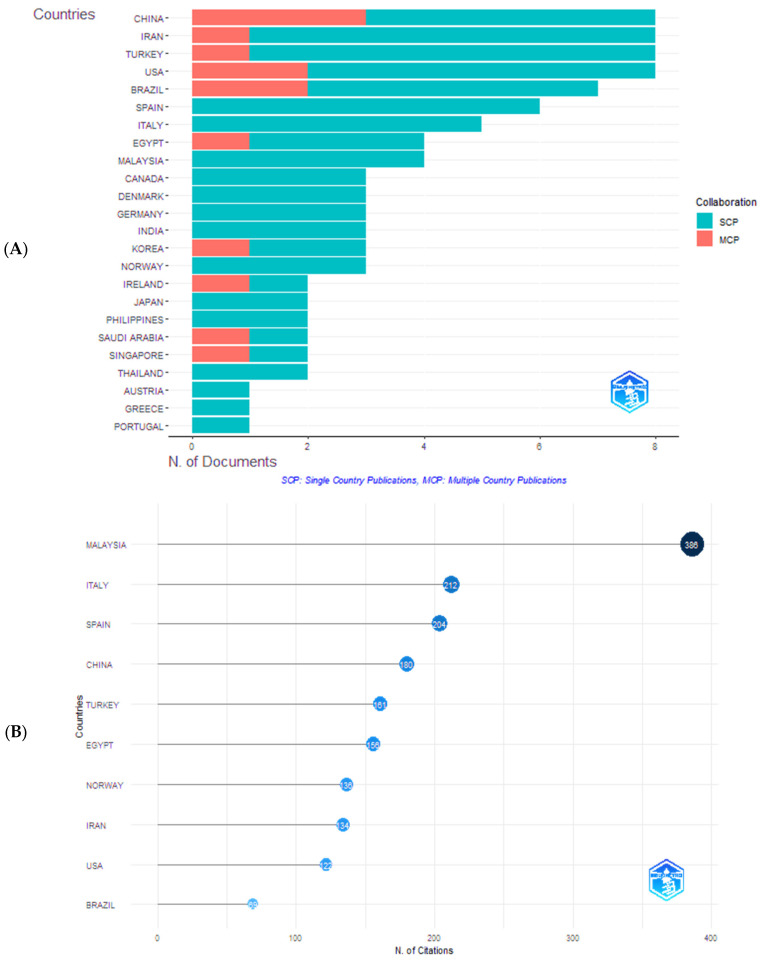
Production by countries of the corresponding author (**A**) and the 10 most cited countries from 1995–2024 (**B**) of analyzed data from the bibliometric analysis of the use of organic acids in aquaculture.

Unlike the scientific production of countries, where an article can be counted multiple times if there are authors affiliated with institutions from different countries, the scientific production by country of the corresponding author counts the documents only once, regardless of the number of co-authors from other countries. Through Figure 8A, a reduction in the scientific production of each country is observed. These are divided into two groups: articles published by authors from a single country, abbreviated as SCP (Single Country Publications), shown in orange, and articles published by authors collaborating with other countries, abbreviated as MPC (Multiple Country Publications), shown in blue.

China, Iran, Turkey, and the United States each published eight articles in the projection of corresponding countries for each author (Figure 8A), dividing the number of articles published by authors from a single country and the number of articles published in collaboration with other countries. China published five independent articles and three in collaboration with other countries. Iran and Turkey published seven articles independently and one with a global contribution each. The United States published six articles without collaborating with other countries and two with global contributions.

Brazil ranks fifth among the 10 countries with the highest number of publications. Of seven articles published on the topic, five were published independently, and two had the collaboration of other countries. Spain and Italy published six and five articles, respectively, without the participation of authors from other countries. Egypt published three articles without collaboration and one article with global contribution. The ninth and tenth positions are Malaysia and Canada, with four and three articles, respectively, published without contributions from other countries.

The analysis of scientific production by country reveals that although nations such as China, Iran, and Turkey have a high volume of independent publications, international collaboration plays a crucial role in expanding scientific impact, as observed in countries like the United States and Brazil, which have a more balanced ratio between independent and collaborative publications. This scenario suggests that while some countries have the infrastructure and funding to conduct research autonomously, global cooperation can accelerate innovation and strengthen knowledge dissemination, fostering the sustainable development of aquaculture. Furthermore, international collaboration plays a fundamental role in the development of human resources, especially considering that educational institutions conduct most research. These partnerships provide researchers with academic and professional experiences in international contexts, promoting the exchange of knowledge, developing new skills, and expanding their network of contacts in different cultural and scientific environments [76].

The relevance of the countries was also measured by the citations attributed to them. The 10 most cited countries are presented below in Figure 8B, and it can be concluded that there is not much similarity between the number of articles published by country and the citations received by each. Regarding the number of publications, Malaysia was in ninth place. Still, it had the highest citation index, occupying the first position with 386 citations, corroborating the results obtained in the present work regarding the most cited authors (Figure 6). An analysis of the database reveals that the average age of articles from Malaysia is higher. For example, the most cited article (144 citations) was published by Ng et al. [64]. This is further reinforced by these studies being published in high-impact journals, such as Aquaculture. Moreover, Malaysia has shown increasing interest in innovation, the adoption of molecular tools, and sustainable aquaculture practices [77]. These factors contribute to the global relevance of Malaysian studies and may explain the high number of citations despite the lower volume of publications.

Italian authors occupy the seventh position regarding the number of articles published, but they are the second most cited, with 212 citations. Iranian authors had the highest number of publications; however, they occupy the eighth position regarding the number of citations: 134. Articles published by Spanish authors jumped from sixth to third place, with 204 citations, followed by Chinese authors, who fell from first to fourth place, with 180 citations. Although China holds the leading position regarding the number of publications, its lower citation rate may reflect structural limitations within its academic publishing system. One of the factors that may contribute to the lower number of citations, compared to other countries, is the publication of studies in local journals with limited international visibility, often due to language barriers and editorial preferences favoring high-impact content published in English. Regardless of scientific quality, these factors can reduce Chinese research’s global reach and citation potential [78]. An illustrative example is the study conducted by Deng et al. [79], which, despite having been published nearly a decade ago, has received only two citations—a fact that may be directly linked to the publication being in Chinese. Another factor that may help explain this low citation frequency is the restricted access to specific publications, such as articles published in journals like Aquaculture, Food Research International, Aquaculture Research, Food Chemistry and Food Control.

Following them are Turkey and Egypt, with 161 and 156 citations, respectively. Norway, which was not among the top 10 producing countries (it held the 15th position, with three published articles), enters the ranking of the 10 most cited countries in the seventh position, with 136 citations. The United States dropped from fourth to ninth place, receiving 122 citations. Finally, Brazil, which held the fifth position with seven published articles, remained among the 10 most cited but occupied the tenth position with 69 citations.

It is worth noting that, in addition to restrictions on antibiotic use, the rise in scientific production during this period can be attributed to factors such as advances in precision livestock farming, which introduced various intelligent systems capable of automating processes, controlling environmental variables, and continuously monitoring animals and their habitat, thereby enhancing efficiency and sustainability in animal production [80]. Another factor may be related to the World Health Organization (WHO) statement that animal-based foods are the best source of high-quality nutrients for children aged 6 to 23 months, resulting in funding initiatives aimed at sustainability, as well as the international demand for animal-based products, boosting the industry and scientific exploration in this field [81].

### 3.7. Most Relevant Institutions

A detailed analysis of the authors’ affiliations is essential to understand the contribution of these institutions to research in a specific area. The results show that a total of 188 institutions participated in the dissemination of these 96 documents.

**Figure 9:**
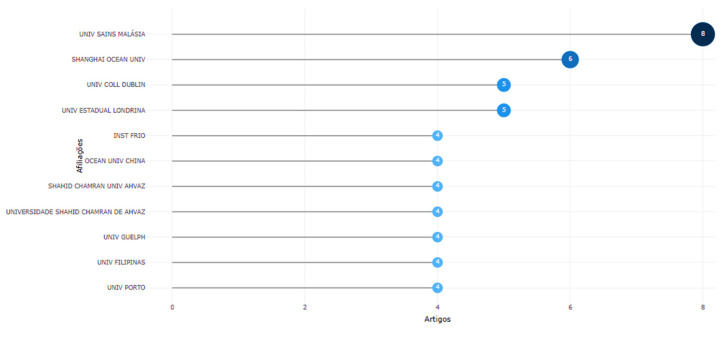
The 10 most relevant affiliations for publishing articles of analyzed data from the bibliometric analysis of the use of organic acids in aquaculture from 1995–2024.

Universiti Sains Malaysia (Public University of Malaysia) was the institution with the most significant contribution to disseminating these documents, publishing eight on the subject (Figure 9). The first publication was in 2009 (two articles); they then published again in 2015 (four articles) and 2016 (two articles), corroborating the results previously described in this work for authors and countries most relevant to the area.

Next were the second and third most relevant institutions, Shanghai Ocean University and the University of Bologna, which each published six articles on using organic acids in aquaculture in 2022, corroborating the results discussed in subitem 3.6.

In fourth place, University College Dublin (University of Dublin) contributed by publishing five articles. This university appears on the timeline in 2018 with two articles and in 2020 with three articles. Figure 9 shows the other affiliations among the top 10 most relevant institutions and their productions over time.

The contribution of the State University of Londrina (from Brazil) appears only in 2023, with the publication of five articles. The contribution of the fifth place, Instituto del Frío (CSIC), appears on the timeline with its first publication in 2006 (two articles), publishing again in 2007 (two articles), and not publishing any more articles related to the topic. The sixth place was the Public University in Qingdao (Ocean University of China), which published its four documents in 2018, and the seventh place published its four documents in 2024 the Iraqi University in Ahvaz (Shahid Chamran University of Ahvaz).

The University of Guelph contributed for the first time by publishing four documents related to the topic in 2021. The documents published by the institution in second-to-last place, the University of the Philippines, appear on the timeline in 2018; all four articles published by this affiliate relate to the subject. Finally, the University of Porto, ranked tenth in the most relevant category by the number of publications, contributed with the publication of four articles released in 2024.

### 3.8. Research Trends

Research is increasingly focused on using organic acids in aquaculture as dietary supplements. The trend is to explore the effects of distinct acids, such as citric, acetic, lactic, ascorbic, propionic, and butyric, either individually, combined, or with other treatments, in various species (Table 7), as well as in different approaches regarding performance, health, disease resistance, and productivity. Analyses on the antibacterial activity of these compounds tend to include new treatments and specific dosages, based on studies that have already proven effective.

**Figure 10:**
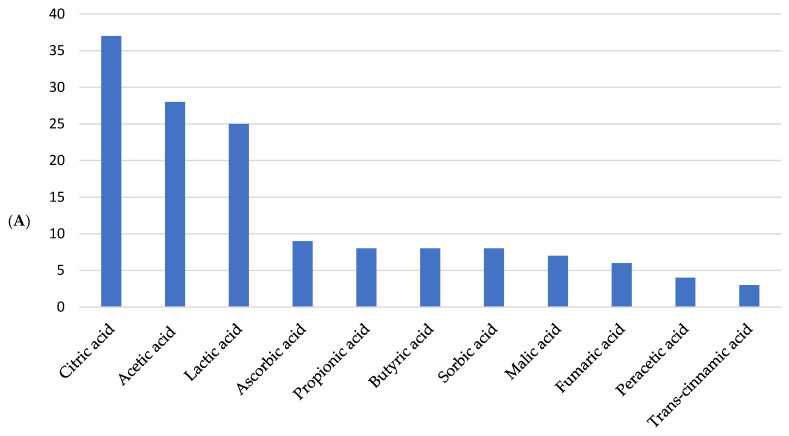

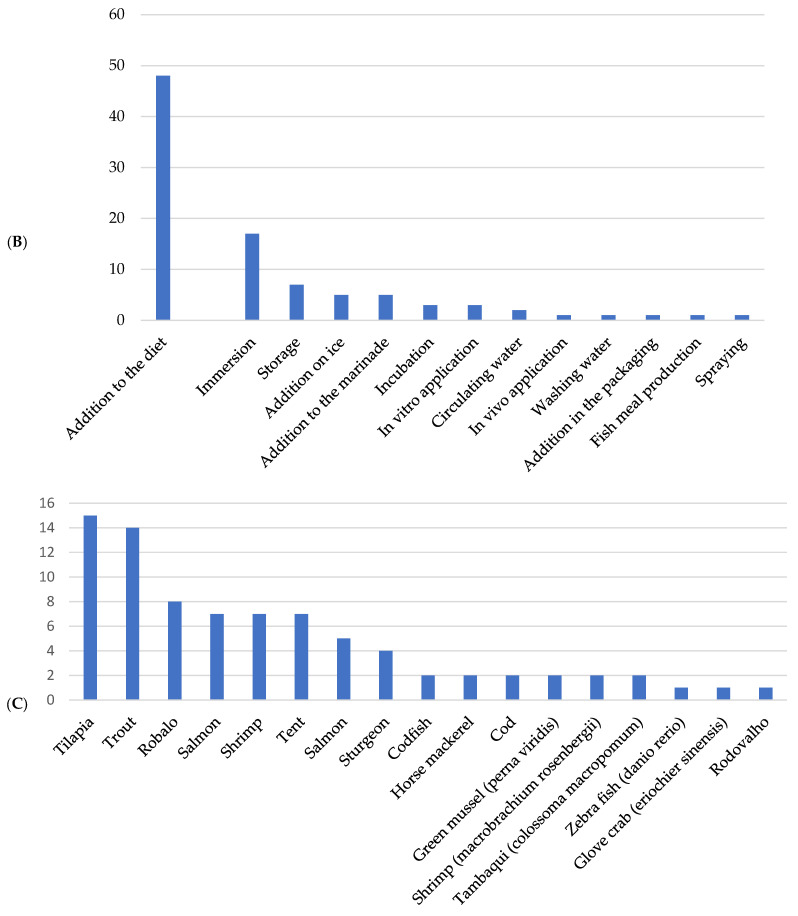
Frequency of treatment types (**A**), organic acids (**B**), and fish types (**C**) of analyzed data from the bibliometric analysis of organic acids in aquaculture.

Figure 10 shows the frequency of types of acids, application forms, and products reported by studies presented in Table 7.

Specific acids can serve as energy sources in metabolic processes, enhancing meat quality. The positive effects of acids result from their interactions, as acid blends are often utilized, making it challenging to identify a single dominant mode of action. These mixtures can have cascading effects, acting as non-antibiotic growth promoters and reducing the excretion of environmentally harmful compounds. However, outcomes have been inconsistent, depending on dosage, treatment duration, acid type, diet composition, animal age, and environmental conditions [101].

Acids also influence the aroma and taste of feed. Ions produced by the dissociation of acids stimulate transient receptor potential channels in the oral cavity, responsible for sensations such as burning and pain, which send signals to the brain. The sour taste is detected by sensory cells in the taste buds, transmitting the stimulus to the brain via cranial nerves. The correct dosage and type of acid can enhance the flavor and aroma of feed; however, incorrect amounts may cause somatic pain and reduce feed intake [102].

Figure 10B demonstrates the application types for the acids found in the studies presented in Table 7. It was observed that most of the studies incorporated organic acids directly, either in meat or in the diet of aquatic animals. The challenges of this technique are, especially, the alteration of sensory characteristics of either the meat or the diet for aquatic animals and the lack of uniformity in the application. However, when direct applications by droplets are used, such as spraying or nebulization, these problems can often be reduced [16].

Another pertinent factor is that the efficacy of acids as additives depends on genotype. Figure 10C demonstrates the types of aquatic animals tested with the acids found in the studies shown in Table 7. Recently, Naya-Català et al. [50] conducted an experiment supplementing organic acids, phytogenics, and probiotics in a feeding trial with reference gilthead sea bream and those selected for growth within the Spanish National Breeding Program (PROGENSA). The authors reported that phytobiotics primarily influenced the transcriptome of genetically selected fish, whereas organic acids modified the gut microbiota of reference fish. Probiotics exhibited an opposite pattern to the other two treatments regarding weight gain and induced changes in both groups’ transcriptome and gut microbiota. These findings suggest that incorporating metagenomics and transcriptomics is crucial for the success of innovative breeding and nutrition programs.

Recent studies have indicated that poor water quality management can significantly harm Nile tilapia (*Oreochromis niloticus*) health, leading to decreased growth performance, altered body composition, compromised antioxidant and immune responses, and changes in physiological and histopathological parameters. While dietary supplementation with organic acids offers enhanced growth and increased disease resistance, its efficacy depends on maintaining optimal water quality. Deterioration in water quality may render organic acids an additional stressor for the fish [103]. Contradictorily, under low dissolved oxygen conditions, organic acids improved Nile tilapia’s growth performance, digestibility, and intestinal morphology [104]. These findings highlight a variability in the results that requires further investigation.

The effects of organic acid supplementation in different production systems can be positive, although they may target distinct objectives. Neves et al. [105] used earthen ponds to raise juvenile Nile tilapia (*Oreochromis niloticus*) for 35 days with diets containing varying concentrations of fumaric acid. The study reported improvements in growth performance, reductions in pathogenic bacterial load, and positive changes in intestinal morphology. In contrast, De Souza e Silva et al. [106] experimented with a controlled recirculating aquaculture system (RAS) to evaluate the effects of a commercial organic acid blend on immune parameters and resistance to the pathogen *Francisella orientalis*. In this case, fish supplemented with 0.5% of the additive showed lower feed conversion ratios, increased lysozyme bactericidal activity, and a significant reduction in mortality following bacterial challenge.

The European Union regulates the use of specific organic acids as technological additives in animal feed and imposes restrictions to prevent indiscriminate use. Some acids, such as acetic acid, irritate the skin when diluted and become corrosive at higher concentrations, as with formic acid. Lactic acid is irritating to the eyes, corrosive to the skin, and can irritate the respiratory tract. Propionic acids are corrosive to the skin, mucous membranes, and eyes [40].

Another factor to consider is the change in production cost with adding acidifiers to the feed. Few studies address the additional cost of these compounds, which is a limitation. Abdel-Tawwab et al. [30] used a commercial product that combines formic, lactic, and citric acid in a polyculture system and, despite the increase in feed costs, supplementation of 3.0 g/kg of diet increased economic returns by 23.3%, mainly due to the high sales of tilapia and the increased weight gain of supplemented animals. However, to optimize the positive effects of these compounds in some cases, it is necessary to use higher doses per ton, and increased supplementation becomes a limiting factor due to the increase in the final cost of the feed [107].

Although these compounds may represent an additional cost compared to conventional ingredients, studies indicate that benefits such as improved zootechnical performance, reduced mortality, and lower disease incidence can offset this investment over the production cycle. According to the findings of Bhujel et al. [107], the supplementation of tilapia diets with organic acids contributes to a positive economic return, mainly due to the reduced use of antibiotics and the observed increases of 3.4-fold in specific growth rate, 1.6-fold in daily weight gain, and 2.1-fold in protein efficiency ratio.

Menanteau-Ledouble et al. [108] also calculated the Return on Investment (ROI) based on the results of supplementing a commercial product, Biotronic^®^ Top3, in a feeding trial with rainbow trout (*Oncorhynchus mykiss*). The authors considered both the reduction in mortality and the cost of the supplement and found an ROI of 0.23, suggesting that the product is economically profitable. In this context, it is recommended to conduct cost-benefit analyses tailored to the specific conditions of each production system before implementation. Developing more effective and economically viable formulations and integrating them with other functional additives may enhance economic feasibility and promote the sustainable large-scale use of these compounds.

Using organic acids in aquaculture has emerged as a promising strategy for optimizing zootechnical performance and enhancing animal health and well-being [46,109]. These compounds also contribute to food safety due to their antimicrobial action [110] and minimize environmental impacts when used as substitutes for antibiotics [31], helping to reduce microbiological contamination and mitigate the release of residues associated with these drugs. However, the lack of standardization in tests and methodologies for applying organic acids compromises the replicability of studies and the comparison of results, making their commercial adoption more effective.

The action of these compounds on zootechnical parameters optimization of animal health and well-being is not entirely established due to the lack of uniformity in the tests. Significant improvements in zootechnical performance were observed in some species [51,65,107,111], while other studies reported no significant difference between treatments and control [29,67,112]. The gut microbiota was positively modulated, promoting a favorable microbial balance in some species [113], but at high concentrations, it could negatively impact the microbiome [46].

Few studies have directly compared the impacts of similar treatments on the gut microbiota of different fish species. Research comparing similar doses and dietary complexes found different results, with the diversity and composition of the intestinal microbiota in some cases not showing significant differences in fish fed increasing doses [114]. Differently, Busti et al. [115] reported non-linear effects on microbial diversity, with an initial reduction followed by an increase at the highest dose. These varied findings make understanding treatment effects across different taxonomic groups difficult.

In in vivo trials, this variability was evident through different concentrations of fumaric acid in distinct fish species, compromising the comparability across studies. For instance, Omosowone et al. [116] tested this compound in the diets of *Clarias gariepinus* at concentrations ranging from 0.05% to 0.2%. In contrast, Neves et al. [24] applied higher levels, from 0.5% to 3%, in feeds formulated for *Oreochromis niloticus*. In the former, including 0.1% led to weight gain without adverse effects on carcass composition, and the 0.05% dose improved hematological parameters. In contrast, the latter study identified 1.5% as the optimal concentration to enhance zootechnical performance and reduce the presence of Gram-negative bacteria.

The use of organic acids as feed additives in aquaculture has emerged as a promising alternative to antibiotic growth promoters (AGPs), particularly in light of increasing regulatory pressure and the demand for more sustainable practices. Combining organic acids with other additives, such as essential oils, may enhance immune responses and gut health. Addam et al. [117], in a study with Nile tilapia (*Oreochromis niloticus*), evaluated dietary supplementation with organic acids combined with the essential oil *Lippia origanoides*. Although no significant effects on growth or feed conversion were observed, the authors reported improved gut health, survival rate, nutrient absorption, and intestinal microbiota.

The effects of interactions between organic acids (trans-cinnamic acid) and probiotics (*Bacillus subtilis*) have also proven to be highly beneficial for the health of rainbow trout. The combination of these components in the diet significantly enhanced the innate immune response, including increased phagocytic activity, respiratory burst activity, and granulocyte percentage and the activity of enzymes such as myeloperoxidase and antiprotease. Furthermore, the combined supplementation improved fish resistance to pathogenic challenge with *Yersinia ruckeri*, resulting in higher survival rates and elevated antibody titers [54].

These findings suggest that specific combinations may offer more effective strategies beyond the isolated use of feed additives. However, well-designed comparative trials with robust experimental designs are still needed to distinguish each compound’s individual and synergistic effects. Within this context, probiotics and essential oils stand out as promising alternatives in aquaculture. In a recent study, the inclusion of 0.015% probiotic (Klu-zetar^®^) in fish diets resulted in the highest weight gain (46.99 g), the best feed conversion ratio (FCR = 1.27), and the highest average daily gain (1.56 g/day), outperforming even the groups supplemented with essential oils alone or in combination [118].

Seafood preservation is critical for the food industry because consumers demand fresh and minimally processed products [119]. Fresh and healthy fish are generally sterile, as their immune system prevents bacterial growth in the meat. However, after death and collection, and during storage, microorganisms invade the meat by moving between the muscle fibers [120]. This occurs because fish have intrinsic and extrinsic characteristics favorable to microbial growth, such as high nutritional composition, high water content, and favorable pH [121].

The fishing industry faces significant challenges in maintaining the quality of fish and seafood, often due to the long distances between capture or production sites and consumers. This factor provides opportunities for microbial growth and recontamination [122]. In addition to contamination by natural microbiota, fish can be easily contaminated during processing, either by spoilage microorganisms or pathogens, becoming asymptomatic hosts and facilitating cross-contamination throughout the industrialization and marketing stages [123]. This problem is aggravated by inadequate handling and the high capacity of microorganisms to form biofilms, representing an excellent risk for the food industry [124].

In addition to microbial growth, food quality degradation is also associated with chemical processes, with lipid oxidation being one of the primary mechanisms. Lipid oxidation can form compounds responsible for rancidity, discoloration, and accumulation of potentially toxic substances harmful to human health [125]. Therefore, to ensure the safety and quality of fish and seafood, protecting them against bacteria, fungi, and other contaminants is essential. The primary methods used to extend the shelf life of these products include suppressing lipid oxidation and preventing bacterial growth [126,127].

To minimize contamination and degradation, innovative techniques, in addition to conventional processes, have shown the potential to improve food safety and quality [128]. In this context, food additives and preservatives are widely used to create barriers against microbiological growth and extend shelf life, with organic acids being a potential additive.

Organic acids as food antimicrobials have also proven effective due to their antibacterial action. These acids can reduce the meat’s pH and permeate the bacterial membrane in their undissociated form [129]. Inside the bacteria, where the environment is more basic, they undergo ionic dissociation, acidifying the cytoplasm, which directly interferes with bacterial metabolism and, consequently, leads to the death of the bacteria [130]. However, further investigation into the long-term effects of organic acid supplementation is needed, as spoilage bacteria, yeasts, and fungi such as *Alicyclobacillus acidoterrestris* and *Aspergillus niger* have developed resistance to organic acids [130,131]. Furthermore, studies on the physiological responses of supplemented animals and the potential long-term environmental impacts of these compounds remain necessary.

In addition to the issue of resistance, methodological variability in preservation processes is a frequently cited critical factor. Applying flake ice systems supplemented with organic acids illustrates this challenge, as it hinders consistent comparisons across post-harvest preservation methods. García-Soto et al. [95] evaluated the efficacy of citric and lactic acids at concentrations ranging from 0.075% to 0.175% and 0.050%, respectively, in preserving European hake (*Merluccius merluccius*). In contrast, Sanjuás-Rey et al. [97] investigated the effects of a formulation containing not only citric and lactic acids but also ascorbic acid (0.08% and 0.04%) in three species: hake, megrim (*Lepidorhombus whiffiagonis*), and monkfish (*Lophius piscatorius*). Microbial inactivation levels varied considerably: reductions greater than 2 log CFU/g (colony-forming units) were observed in the first study, whereas in the second, final mesophilic counts remained below 7 log CFU/g for hake and megrim and below 6 log CFU/g for monkfish.

In this context, it is equally relevant to consider the guidelines that govern the application of these compounds, particularly within food systems. Commission Regulation (EU) No. 231/2012 of 9 March 2012 establishes the specifications for food additives, including organic acids commonly used in the food industry and aquaculture, such as malic acid (E 296), fumaric acid (E 297), ascorbic acid (E 300), benzoic acid (E 210), acetic acid (E 260), and lactic acid (E 270). This regulation provides detailed specifications for food additives, covering their definition, chemical composition, purity criteria, and contaminant limits.

Lactic and acetic acids are also subject to regulatory restrictions, such as those established by the European Food Safety Authority (EFSA). The agency authorizes using both as decontamination agents but sets safe concentration limits: 2% to 5% for lactic acid and 2% to 4% for acetic acid. These compounds can be applied by spraying onto carcass surfaces or by spraying and immersing (for 5 to 30 s) during the processing of pork cuts [132].

The Food Safety and Inspection Service [133], an agency within the United States Department of Agriculture (USDA), maintains a repository of technologies primarily focused on meat and poultry processing. FSIS has approved the use of 2.5% citric acid as an antimicrobial agent in the processing of cattle heads. Additionally, it has authorized the use of an aqueous solution containing up to 400 ppm of peroxyacetic acid and 280 ppm of hydrogen peroxide for microbial control in carcasses, parts, trimmings, and organs of cattle or swine, and up to 1000 ppm of peroxyacetic acid and 690 ppm of hydrogen peroxide in parts, organs, and carcasses of poultry. The solution may optionally include acetic acid or sulfuric acid, depending on the pH required for the water used in washing and cooling processes.

Another relevant approach in this context is the microencapsulation of organic acid mixtures, which aims to improve the efficiency of dietary supplements and promote better zootechnical performance. Studies on the combined effects of different acids in various aquaculture branches, such as fish farming, shrimp farming, and malacoculture, represent a promising area of research [134].

The emphasis on sustainable practices is also relevant to using organic acids in aquaculture, as these compounds improve animal health and welfare and reduce environmental impacts. Reducing microbiological contamination and improving waste management are additional benefits that reinforce the importance of these additives for the sector’s sustainability.

## 4. Conclusions

The results of the bibliometric analysis demonstrated the growing interest in aquaculture activities, which has led to a significant expansion of scientific production in the field. China has stood out in this context, contributing the most published articles over the past 30 years. Organic acids in aquaculture have received global attention primarily as a dietary supplement, effectively reducing microbial loads in closed culture and water reuse systems. The addition of these acids to the diet of groups of species, such as red hybrid tilapia, described in the most cited article of this bibliometric analysis over a decade ago, already projected the importance of organic acids in improving aquaculture health and productivity.

In the studies conducted, among the species investigated, the ones that stood out the most were tilapia (*Oreochromis niloticus*), rainbow trout (*Oncorhynchus mykiss*), European sea bass (*Dicentrarchus labrax*), common carp (*Cyprinus carpio*), Siberian sturgeon (*Acipenser baerii*), Atlantic salmon (*Salmo salar*), and whiteleg shrimp (*Litopenaeus vannamei*). The report made in 2016 in the third most referenced article (here cited as [34]), according to this bibliometric analysis, corroborated the assertion that acidifiers improved growth performance, blood parameters, and body composition of tilapia, increasing disease resistance, confirming the antibacterial activity of the acids in vitro and in vivo.

The research covered in this review’s second-most-cited article reaffirms the effectiveness of organic acids in fish and shrimp farming. Evidence points to positive impacts on different shrimp species, including improvements in zootechnical performance, modulation of the intestinal microbiota, increased digestibility, and greater resistance to pathogens. These findings reinforce the potential of organic acids as a promising strategy to optimize the health and performance of organisms grown in aquaculture.

Based on the analyzed studies, the most frequently mentioned acids, such as citric, acetic, lactic, ascorbic, and propionic acids, have demonstrated various applications in aquaculture. These acids were manipulated during feed production to prevent contamination and used as dietary supplements, incorporated into refrigeration ice, applied in immersion and spraying decontamination treatments, and utilized as food preservatives and pathogen inactivators, both in vivo and in vitro. These results highlight the versatility and potential of organic acids as practical tools in improving safety and quality in aquaculture.

In summary, using organic acids in aquaculture is a promising strategy to promote sustainability, enhance fish health and performance, and reduce environmental impacts. However, their effectiveness depends on dosage, acid type, fish genotype, and environmental conditions, requiring tailored and integrated approaches. Future research should investigate organic acids’ species-specific dose–response relationships under different environmental conditions, to reflect real-world production systems. Studies should also focus on evaluating organic acids individually, rather than exclusively in combination with other functional additives. Moreover, further investigation is needed regarding the long-term effects on fish physiology and overall health and the environmental impacts and economic viability associated with their use.

While regulatory barriers are essential to minimizing risks to human and animal health and preventing the indiscriminate use of potentially harmful compounds at high concentrations, they can also hinder the large-scale adoption of organic acids as effective alternatives. This, in turn, slows technological innovation and limits the expansion of their applications. Despite these challenges and occasional inconsistencies, organic acids remain valuable supplements for aligning productivity, sustainability, and animal welfare.

## Figures and Tables

**Table 1:** Overview of key information from the bibliometric analysis of using organic acids in aquaculture.

Main Information About the Data	Results
Timespan	1995:2024
Sources (Journals, Books, etc.)	44
Documents	96
Annual Growth Rate %	8.26
Document Average Age	6.45
Average Citations Per Doc	22.47
References	2285
Document Contents	
Keywords Plus (ID)	737
Author’s Keywords (DE)	317
Authors	
Authors	426
Authors of Single-Authored Docs	1
Authors Collaboration	
Single-Authored Docs	1
Co-Authors Per Doc	5.79
International Co-Authorships %	14.58
Document Types	
Article	93
Article: Early Access	1
Article: Proceedings Paper	2

**Table 2:** The 10 leading sources of local impact by H-index of analyzed data from the bibliometric analysis of using organic acids in aquaculture.

Element	H_Index	TC	NP	PY_Start
Aquaculture Research	8	373	8	2009
Aquaculture	7	291	14	2015
Journal of Food Protection	5	90	5	1995
Aquaculture International	4	57	4	2011
Aquaculture Reports	3	15	3	2023
International Journal of Food Microbiology	3	136	3	2009
Journal of Food Science	3	53	3	2010
PeerJ	3	146	3	2018
Animals	2	16	3	2022
Aquaculture Nutrition	2	74	3	2018

TC: total citations; NP: number of publications; and PY Start: initial year of publication analysis.

**Table 3:** The 10 authors with the highest impact factor by H-index of analyzed data from the bibliometric analysis of using organic acids in aquaculture.

Element	H_Index	TC	NP	PY_Start
Koh C	4	386	4	2009
Aubourg S	3	81	3	2012
Martins M	3	37	3	2019
Meinelt T	3	67	3	2012
Ng W	3	317	3	2009
Pereira S	3	37	3	2019
Sahu N	3	30	3	2006
Santos G	3	34	3	2017
Yilmaz S	3	130	3	2018
Abedian K A	2	57	2	2018

TC: total citations; NP: number of publications; and PY_Start: initial year of publication analysis.

**Table 4:** Number of articles published by authors and frequency according to Lotka’s Law of the analyzed data from the bibliometric analysis of the use of organic acids in aquaculture.

No. Articles	No. Authors	Freq
1	309	0.72535211
2	105	0.24647887
3	11	0.0258216
4	1	0.00234742

**Table 5:** The top 10 most globally cited documents of analyzed data from the bibliometric analysis of the use of organic acids in aquaculture.

Paper	DOI (Digital Object Identifier)	Reference
Ng W. K., 2009, Aquac Res	10.1111/J.1365-2109.2009.02249.X	[64]
Romano N., 2015, Aquaculture	10.1016/J.Aquaculture.2014.09.037	[42]
Reda R. M., 2016, Fish Shellfish Immunol	10.1016/J.Fsi.2016.01.040	[65]
Elvira Lopez-Caballero M., 2007, Int J Food Sci Technol	10.1111/J.1365-2621.2006.01328.X	[66]
Qiu X., 2014, Food Chem	10.1016/J.Foodchem.2014.04.037	[68]
Rimoldi S., 2018, Peerj1	10.7717/Peerj.5355	[67]
Koh C. B., 2016, Aquac Res	10.1111/Are.12492	[51]
Schirmer B. C., 2009, Int J Food Microbiol	10.1016/J.Ijfoodmicro.2009.05.015	[69]
Yilmaz S., 2018, J Appl Microbiol	10.1111/Jam.14097	[54]
Ng W. K., 2015, Aquaculture	10.1016/J.Aquaculture.2015.02.006	[43]

**Table 6:** Scientific production of the top 10 countries of analyzed data from bibliometric analysis of the use of organic acids in aquaculture.

Region	Frequency
China	26
Iran	25
USA	21
Brazil	20
Turkey	20
Egypt	18
Italy	16
Spain	12
Malaysia	11
India	10

**Table 7:** Main organic acids used with their respective treatments and aquatic species of analyzed data from the bibliometric analysis of organic acids in aquaculture (OA: organic acids).

Acid Types	Treatments	Species of Fish	Reference
Combination of OA	Addition to the diet	Tilapia (*Oreochromis* sp.)	[6,51]
Citric	Addition to the diet	Yellow tail (*Seriola quinqueradiata*); Rodovalho (*Scophthalmus maximus* L.)	[53,82]
Citric	Immersion	Shrimp mantis (*Erugosquilla massavensis*); Barramundi (*Lates calcarifer*); Robalo (*Lateolabrax japonicas*)	[68,83,84]
Acetic	Addition to the diet	Pacific white shrimp (*Penaeus vannamei*); Siberian sturgeon (*Acipenser baerii*)	[85,86]
Acetic	Storage	Smoked salmon (*Salmo salar*)	[87]
Lactic	Addition to the diet	Common carp (*Cyprinus carpio*); Beluga sturgeon (*Huso huso*)	[88,89]
Lactic	Immersion	Lagoon mullet (*Mugil cephalus*); Catfish (*Pangasianodon*)	[90,91]
Lactic	Storage	Sardine (*Sardina pilchardus*); Lagoon mullet (*Mugil cephalus*)	[90,92]
Lactic	Immersion of storage	Green mussel (*Perna viridis*); Codfish (*Gadus morhua*)	[93,94]
Citric and lactic	Addition on ice	Cod (*Merluccius merluccius*)	[95]
Acetic, citric, and lactic	Immersion	Fish meat	[96]
Lactic, citric, and peracetic	Immersion	Golden (*Sparus aurata*)	[33]
Citrus, ascorbic, and lactic	Addition on ice	Horse mackerel (*Scomber scombrus*); Cod (*Merluccius merluccius*)	[97,98]
Ascorbic, citric, and lactic	In vitro	Salmon (*Salmo salar*); Codfish (*Gadus morhua*)	[99]
Citrus, ascorbic, and acetic	Spraying	Norwegian lobster (*Nephrops norwegicus*)	[100]

## Data Availability

No new data were created or analyzed in this study.
